# Heart Failure—Focus on Kidney Replacement Therapy: Why, When, and How?

**DOI:** 10.3390/ijms26062456

**Published:** 2025-03-10

**Authors:** Ewa Wojtaszek, Marlena Kwiatkowska-Stawiarczyk, Małgorzata Sobieszczańska-Małek, Tomasz Głogowski, Aleksandra Kaszyńska, Michał Markowski, Sławomir Małyszko, Jolanta Małyszko

**Affiliations:** 1 Department of Nephrology, Dialysis and Internal Diseases, Medical University of Warsaw, 02-097 Warsaw, Poland; wojtaszek.ewa@gmail.com (E.W.); marlenakwiatko@gmail.com (M.K.-S.); tomglogowski@gmail.com (T.G.); ola.kaszynska@gmail.com (A.K.); m.markowski22753@gmail.com (M.M.); 2 Department of Cardiac Surgery, Thoracic Surgery and Transplantation, Medical University of Warsaw, 02-097 Warsaw, Poland; m_sob@poczta.onet.pl; 3 Department of Invasive Cardiology, University Teaching Hospital, 15-276 Bialystok, Poland; smalyszko1999@gmail.com

**Keywords:** heart failure, chronic kidney disease, acute kidney injury, congestion, kidney replacement therapy, extracorporeal ultrafiltration, peritoneal ultrafiltration

## Abstract

Heart failure (HF) is a major health problem because of its high prevalence, morbidity, mortality, and cost of care. An important contributor to morbidity and mortality in patients with advanced HF is kidney dysfunction. Almost half of HF patients develop cardiorenal syndrome (CRS). The management of advanced HF complicated by CRS is challenging. Two main strategies have been widely accepted for the management of CRS, namely improving cardiac output and relieving congestion. Diuretics remain the cornerstone and first-line therapy for decongestion; however, a substantial number of CRS patients develop diuretic resistance. In the face of persistent congestion and the progressive deterioration of kidney function, the implementation of kidney replacement therapy may become the only solution. In the review the current evidence on extracorporeal and peritoneal-based kidney replacement techniques for the therapy of CRS patients are presented.

## 1. Introduction

Heart failure (HF) is considered a global pandemic affecting more than 64 million people and is a growing public health problem worldwide [1,2]. Despite continuous developments in evidence-based therapies, many HF patients will progress to advanced stages of the disease with multiple organ dysfunction [3]. It is estimated that 51% of HF patients have severe HF, and about 5% progress to the end-stage stadium [2]. For such patient, heart transplantation and long-term circulatory support remain the only effective treatment. However, most patients, due to comorbidities and age, are not suitable for such procedures. Due to acute HF decompensation, they require recurrent hospitalizations, and 1-year survival in this population is less than 50% [4,5,6].

Fluid overload and congestion constitute the hallmark of most patients with HF. Acute decompensated heart failure (ADHF), a condition characterized by signs and symptoms of congestion, is associated with an increased risk of multiple organ dysfunction, prolonged and recurrent hospitalization, and mortality [7]. Systemic congestion is a target in the management, and its relief is considered a treatment success [8]. Nevertheless, patients with ADHF frequently experience suboptimal decongestion. Nearly 50% of them leave the hospital with inadequate weight loss, and 24% of them require rehospitalization within 30 days [5,7,9].

An important contributor to morbidity and mortality in patients with advanced HF is kidney dysfunction [3,10]. The bidirectional relationship between the heart and kidneys, whereby the failure of one organ escalates pathological changes in the other, is defined as cardiorenal syndrome (CRS) [11,12]. Almost half of HF patients develop CRS [11,13]. The management of these patients is challenging.

### 1.1. Cardiorenal Syndrome

Ronco et al. described five types of CRS [14]. Type 1 is characterized by abrupt worsening of cardiac function (e.g., acute decompensated heart failure) leading to acute kidney injury. Acute heart failure may be presented as on one of four subtypes, namely hypertensive pulmonary edema with preserved systolic left ventricular ejection function, acutely decompensated chronic heart failure, cardiogenic shock, and predominant right ventricular failure. The early diagnosis of acute kidney injury remains a challenge in type 1 CRS. In our review we will focus on type 1 CRS. Type 2 CRS is characterized by chronic abnormalities in cardiac function leading to chronic kidney disease. In type 1 and type 2, primary heart disorders result in secondary kidney dysfunction/injury. Type 3 CRS consists of a sudden kidney function decline (e.g., acute kidney injury) leading to acute cardiac dysfunction (e.g., heart failure, etc.). In type 4 CRS, chronic kidney disease contributes to decreased cardiac function. In type 3 and type 4, primary kidney disorder contributes to secondary heart dysfunction. In Type 5 CRS, systemic conditions (e.g., sepsis) result in the dysfunction of both organs e.g., the heart and kidneys [12,14]. In our review, we will focus on type 1, in particular how nephrologists may help with the wise and appropriate introduction of kidney replacement therapy.

A plethora of interacting pathophysiological mechanisms contribute to CRS; however, many of them are still not fully understood [11,12,15]. In acute and chronic cardiorenal syndrome (type 1 and 2) reduced cardiac output results in diminished renal perfusion and neurohormonal activation, leading to kidney dysfunction and the development of CRS [15,16,17,18]. Reduced renal perfusion results in the activation of the sympathetic nervous system and the renin–angiotensin–aldosterone system (RAAS), and in the stimulation of the release of arginine vasopressin [15,18]. This later leads to systemic and renal vasoconstriction and increased sodium and water retention to preserve renal perfusion; however, rises in plasma volume contribute to worsening HF [10,15,18]. The activation of the renin–angiotensin–aldosterone system (RAAS) and the sympathetic nervous system is well documented in HFrEF and in a subgroup of patients with HF with preserved ejection fraction (HFpEF) [13,16,17]. On the other hand, it has been postulated that venous congestion, increased central venous pressure (CVP), and increased renal venous pressure contribute to renal dysfunction in CRS [16,17,18,19,20,21]. A rise in CVP leads to neurohormonal activation, promotes inflammation and oxidative stress and causes excessive renal tubular sodium reabsorption, resulting in volume overload [3,11,15,16,17,22,23]. All these events contribute to right ventricle stress, renal congestion, and a rise in intra-abdominal pressure (IAP) [16,17,19,20,21], leading to the exacerbation of HF or the development of acute decompensation of HF. Initially, elevated CVP favors a slight increase in glomerular filtration rate (GFR) through increased glomerular hydrostatic pressure and glomerular hyperfiltration [16,17,21]. The GFR progressively declines due to renal edema, increased interstitial pressure, tubular compression, and intracapsular tamponade [17]. An increase in CVP above 6 mmHg is associated with kidney function impairment. Moreover, increased IAP above 12 mmHg may also contribute to kidney dysfunction [24]. Deterioration of kidney function occurs more frequently in patients with preserved ejection fraction (HFpEF) than reduced ejection fraction (HFrEF), probably due to more pronounced venous congestion [16,17,20,21,24]. It is clinically relevant, as in a congestive state, an impaired response to diuretics/diuretic resistance may result from the physiological phenomena of diuretic braking (diminished diuretic effectiveness secondary to post-diuretic sodium retention) [25] and post-diuretic sodium retention [26].

**Figure 1:**
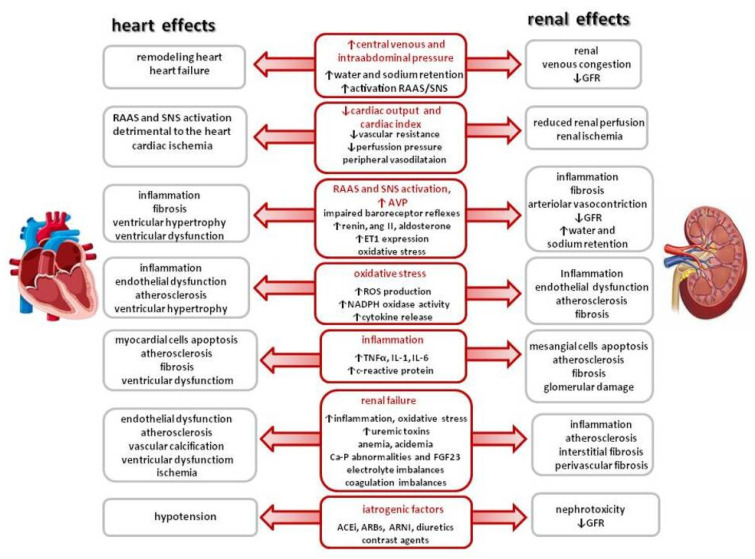
Pathophysiological factors and mechanisms involved in CRS pathophysiology. RAAS—renin–angiotensin–aldosterone system, SNS—sympathetic nervous system, GFR—glomerular filtration rate, AVP—arginine vasopressin, ET-1—endothelin 1, ROS—reactive oxygen species, TNFα—tumor necrosis factor α, IL-1—interleukin 1, IL-6—interleukin 6, FGF23—fibroblast growth factor 23, ACEi—angiotensin-converting enzyme inhibitor, ARB—angiotensin receptor blocker, and ARNI—angiotensin receptor-neprilysin blocker.

In addition, subclinical inflammation, and oxidative stress promote fibrosis and apoptosis in both kidney and heart perpetuating CRS [15,19,22,23]. Moreover, other mechanisms, such as endothelial dysfunction, the involvement of small noncoding RNAs, and epigenetic alterations, may also contribute to the development and progression of CRS [11,24,27,28]. The clinical importance of each mechanism is likely to vary from patient to patient (e.g., pulmonary edema due to hypertension vs. acute cardiogenic shock) and from situation to situation (from acute HF secondary to perforation of a mitral valve leaflet in a course of endocarditis vs. exacerbation of right HF secondary to diuretic therapy resistance). The pathophysiological factors and mechanisms involved in CRS pathophysiology are summarized in Figure 1.

The overall management of CRS is challenging and requires a multidisciplinary approach to tackle the many manifestations and complications, as elegantly described recently [29]. The treatment was not assessed in randomized clinical trials designed for CRS; hence, therapeutic strategies based on evidence from HF trials cannot be recommended to patients with CRS because they are mainly derived from the current therapeutic options based on evidence from HF trials [30]. As no universally accepted algorithms are available for CRS, real-world therapy for CRS is a perfect example of personalized medicine, as treatment options vary substantial from one patient to another. Therapy for CRS predominantly involves hemodynamic stabilization, decongestion using diuretics or kidney replacement therapy if needed, inotropes to improve of cardiac output, and goal-directed medical treatment with the inhibition of renin–angiotensin–aldosterone system, beta-blockers, sacubitril/valsartan, or SGLT2 inhibitors. Recently, the EMPULSE trial (Empagliflozin in Patients Hospitalized with Acute Heart Failure Who Have Been Stabilized) has demonstrated improvements in symptoms and quality of life seen as early as 15 days after initiation of SGLT2i in patients with acute decompensated CHF [31]. In a recent systematic review and network meta-analysis, the efficacy and safety of emerging therapies for heart failure with reduced ejection fraction (HFrEF) were evaluated for specific groups of patients. It has been found that sacubitril/valsartan, SGLT2 inhibitors (sodium–glucose transporter 2), i.e., dapagliflozin, and verciguat reduced the primary outcome composite endpoint of cardiovascular death (CVD) and HF hospitalization (HFH) in patients with chronic kidney disease [32].

**Figure 2:**
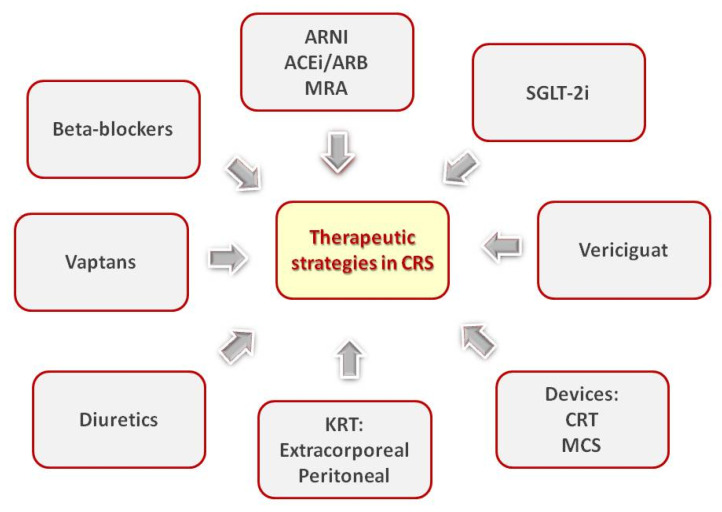
Treatment strategies for cardiorenal syndrome.

Figure 2 presents two main strategies widely accepted for the management of CRS, namely improving cardiac output and relieving congestion.

Diuretics remain the cornerstone and first-line therapy for decongestion. They are recommended as first-level evidence in guidelines; however, robust clinical evidence from clinical trials is limited, and its use is very much empirical [30,33]. There is no evidence on the optimal dosage and the mode of administration [33,34]. In general, diuretic dose should be titrated based on repetitive assessments of the response, including measurement of hourly urine output and spot urine sodium content [34,35]. Recent evidence suggests that intensive diuretic therapy with high doses of loop diuretics is safe, and rapid resolution of congestion may have nephroprotective effect despite temporary increase in serum creatinine [36,37]. So-called “permissive hypercreatininemia” is perceived when adequate diuresis is present because of effective decongestion and is associated with better long-term outcomes [9,38]. Nevertheless, more than 20% of patients with CRS experience failure to decongest despite the escalation of diuretics doses [33]. Several mechanisms are involved in the development of diuretic resistance. They include variations in drug pharmacokinetics affecting drug delivery or drug–drug interactions. Moreover, when GFR is reduced, the reuptake of sodium by tubular cells is downregulated, and chronic administration of loop diuretics additionally reduces the reabsorption of sodium in the loop of Henle and increases sodium delivery to the early distal convoluted tube. This leads to the remodeling of tubular kidney cells and increased sodium reabsorption with decreased natriuresis [39,40,41]. In the case of loop diuretic resistance, a sequential nephron blockade strategy is applied. It involves combination of loop diuretics with other diuretics with different mechanisms of action, including thiazide, thiazide-like agents, acetazolamide, and mineralocorticosteroid receptor antagonists [42,43,44]. Unfortunately, this approach has not been sufficiently evaluated in clinical trials, and in many patients with kidney dysfunction, these combinations are contraindicated or poorly tolerated. In these patients, persistent congestion, especially if accompanied by deterioration of kidney function, carries an elevated risk of poor clinical outcomes [9,36]. It is estimated that almost half of patients hospitalized with ADHF experience worsening kidney function or the development of acute kidney injury. Up to 38% of these patients have underlying chronic kidney disease (CKD) emphasizing the substantial burden of CRS [7,10,25]. In the face of resistance to diuretics and impaired kidney function, the only solution for effective decongestion appears the initiation of use of kidney replacement therapy to affect the course of the disease and/or improve the patient’s quality of life. Kidney injury requiring some form of kidney replacement therapy (KRT) occurs in 1% to 3% of patients hospitalized with heart failure [45,46]. The choice of technique and its prescription require consideration of the needs for fluid or fluid and solute removal, the urgency of decongestion, and patients’ hemodynamic tolerance.

### 1.2. New Concept of Cardiovascular–Kidney–Metabolic (CKM) Syndrome

**Figure 3:**
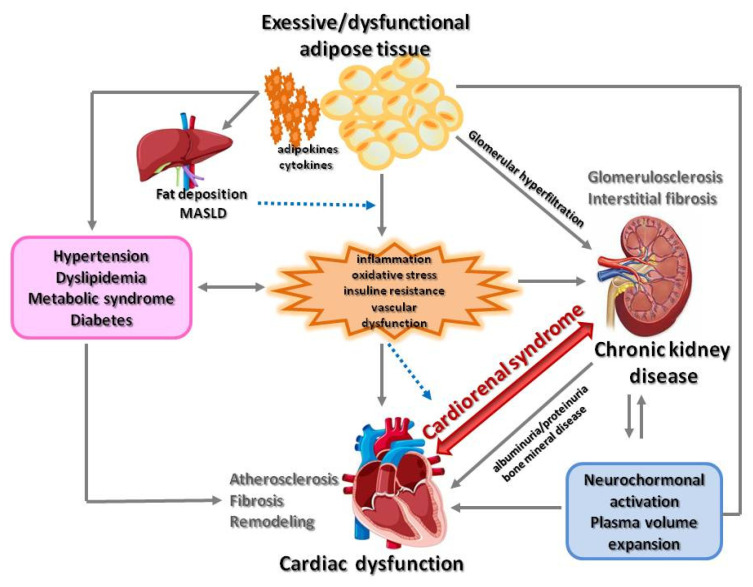
Pathophysiology of cardiovascular–kidney–metabolic syndrome. Based on [47] (MASLD—metabolic dysfunction-associated steatotic liver disease).

In the last year, the concept of cardiovascular–kidney–metabolic (CKM) syndrome was introduced by the American Heart Association [47] This is the clinical presentation of the pathophysiological interactions among metabolic risk factors, such as obesity and diabetes, chronic kidney disease, and the cardiovascular system. The authors stressed that although both cardiorenal and cardiometabolic syndromes are well recognized, there is a growing awareness that metabolic abnormalities play a key pathophysiological role in bidirectional cardiovascular–kidney interactions. Moreover, impaired kidney function is increasingly recognized as a key mediator of the associations between metabolic risk factors and CVD, particularly heart failure (HF) [48] Therefore, taking into consideration the overlap between these two syndromes, the authors devised the concept of a broader construct of CKM syndrome, as shown in Figure 3.

In the short paragraph on stage 4B (CKM with kidney failure) the authors only mentioned the fact that there were limited high-quality data to guide best practices for HF and ASCVD management in kidney failure. They stressed that frequent dialysis sessions to reduce left ventricular hypertrophy/left ventricular mass index and HF hospitalizations and to improve quality of life should be considered [49,50]. Finally, they recommended that an early multidisciplinary approach with advanced HF specialists’ involvement should become the comprehensive CKM care approach for these patients [51]. As this paper is mainly cardiocentric, and there is almost nothing about kidney replacement therapy, except for the above statements, we will instead focus on the cardiorenal syndrome and therapy of both components.

## 2. Kidney Replacement Therapy

**Figure 4:**
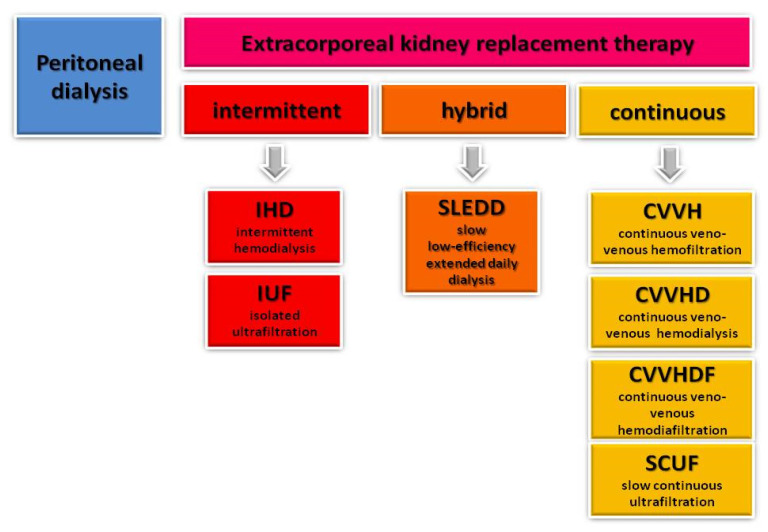
Kidney replacement therapy modalities.

KRT can be delivered as peritoneal dialysis (PD) and various forms of extracorporeal techniques, including intermittent (isolated ultrafiltration (IUF) and intermittent hemodialysis (IHD)), hybrid (slow low-efficiency daily dialysis (SLEDD)), and continuous (slow continuous ultrafiltration (SCUF), continuous veno-venous hemofiltration (CVVHF), continuous veno-venous hemodiafiltration (CVVHDF), and continuous veno-venous hemodialysis (CVVHD)), as shown on the Figure 4. In addition, KRT also ultimately includes kidney transplantation.

**Figure 5:**
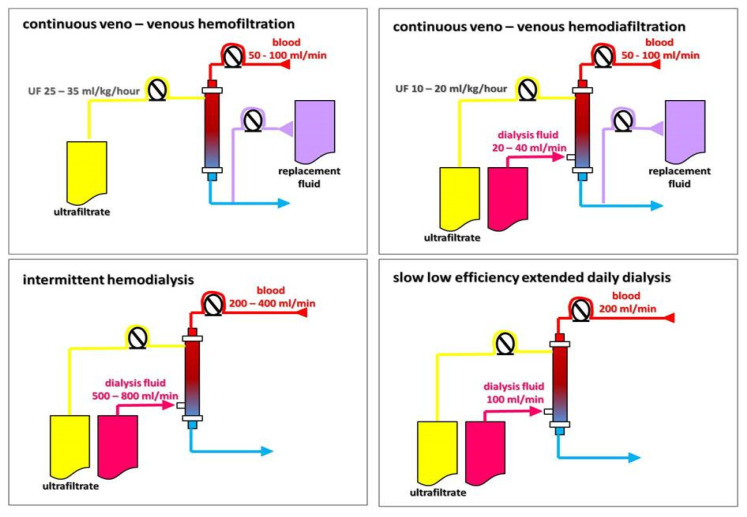
Principles of extracorporeal kidney replacement therapy modalities.

The goals of various KRT techniques are to remove waste products and excess water, balance electrolytes, and replenish buffers in patients with renal failure [52]. KRT procedures differ in one or more of the following qualities: duration of individual treatment sessions, the processes which predominantly mediate the removal of solute and water, and blood and dialysate flow [53,54,55]. Metabolic clearance through a semi-permeable membrane occurs mainly through diffusion and convection, which can be used alone or together. Diffusive clearance is defined by a solute concentration gradient in the blood and dialysate that is maximized by the countercurrent flow of blood and dialysate [52,53,54]. Convective clearance uses a hydrostatic pressure gradient to extract plasma water and low-molecular-weight solutes, leading to ultrafiltration of the isotonic fluid [52,53,54,55]. Different blood purification techniques use diffusion and convection processes to varying degrees. The dominant type of transmembrane transport has a decisive impact on the ability to remove solutes of a specific molecular weight, and, thus, on the effect of the technique used. In intermittent hemodialysis and slow, low-efficiency dialysis, the processes of diffusion and convection (or ultrafiltration) occur simultaneously, but the dominant method of blood purification is diffusion. Diffusion is effective in removing solutes with a molecular weight not exceeding 1000 Da, e.g., urea and creatinine [53,54]. Using the same equipment, the course of the treatment can be programmed in such a way as to turn off the diffusion process, leaving only ultrafiltration. This technique, called isolated UF, is used when only dehydration/decongestion is necessary. Only convection, without diffusion, is the essence of the blood purification process in hemofiltration. Finally, convection with elements of diffusion is the basis for solutes removal during hemodiafiltration. Convection is effective in removing large molecules with a mass over 10,000 Da, e.g., middle molecules or inflammatory cytokines [53,54,55] (Figure 5). In slow low-efficiency extended dialysis, the mechanism of transmembrane transport is diffusion, but by extending the treatment time, the extremely beneficial effect of perfect diffusion is achieved, as in high-efficiency HD, while maintaining the stability of the circulatory system, as in convection techniques; classically, in SLED, the dialysis fluid flow is half as fast as the blood flow. The comparison of different blood purification methods is presented in Table 1.

Traditionally, the decision to initiate KRT in patients with CRS is based on the following standard indications: acidosis, electrolyte derangements, volume overload, and uremia [52]. The evidence to support the choice of intermittent, hybrid, or continuous blood purification techniques in HF patients is scarce. Several trials comparing different modalities were conducted in a broader critical care setting and provide conflicting evidence [56,57,58]. In a meta-analysis of 17 studies including critically ill patients with acute kidney injury, with 31% in a cardiac care unit, the initiation of RRT with SLEDD was not associated with a different clinical course, renal recovery, or survival as compared with CVVHDF [59]. However, hybrid therapy could be a great advantage given satisfactory hemodynamic stability, better patient rehabilitation, and reduced health resources. Nevertheless, techniques based on convective transport may provide additional benefits in patients with CRS due to their ability to remove large molecules, including pro-inflammatory cytokines. In Libetta et al. study, the implementation of intermittent hemodiafiltration (HDF) in patients with refractory HF demonstrated significant removal of fluid, improved diuretic responsiveness, and significant reductions in circulating pro-inflammatory cytokines (interleukin-8-IL-8 and monocyte chemoattractant protein 1-MCP-1) [60]. In a Japanese, prospective study including patients with CRS, with a follow-up period of 24 months, the authors compared the results of CVVHD and SCUF treatment. Overall, survival rates were higher, particularly in the CVVH group in patients with a lower urine output and cardiomyopathy [61]. The authors concluded that CVVH is beneficial, most likely due to the more effective removal of harmful cytokines. The latter conclusion, however, was only speculative because serum cytokine levels were not measured in this study.

There are also some data that intermittent HD may be beneficial in patients with CRS without end-stage kidney disease. In a retrospective cohort study, patients with refractory, NYHA class IV heart failure (HFpEF and HFrEF) and chronic kidney disease (stages 3 and 4) were treated with intermittent hemodialysis. The authors observed better survival of terminal cardiorenal patients treated with hemodialysis than in the general NYHA IV population, with lower hospital readmission rate and shorter length of stay heart failure hospitalizations [62]. A more recent study has also demonstrated that long-term dialysis therapy for volume overload in advanced HF is a feasible and reasonably safe option to reduce HF hospitalizations [63].

## 3. Extracorporeal Ultrafiltration

The most common indication for KRT initiation in HF patients is refractory volume overload. Isolated ultrafiltration is a potential option for the management of refractory CRS with diuretic resistance [33,64]. Ultrafiltrate produced during this process is composed of water and small solutes, such as sodium and potassium, and is almost isotonic compared to plasma. The reduction in intravascular water volume leads to hemoconcentration and increased serum oncotic pressure. It favors the shift of fluid from the interstitial to intravascular compartment. In this way, although fluid is removed only from the intravascular space, it will ultimately lead to the removal of fluid from extravascular compartments [64,65,66]. It is necessary to adjust the ultrafiltration rate to avoid overly rapid fluid removal from the intravascular space with a potential adverse impact on hemodynamics or vital organ perfusion to make this process effective [64,66]. In Table 2, the potential advantages of ultrafiltration in HF are presented.

Isolated UF can be performed with conventional hemodialysis machines, and for years has been a part of daily clinical practice in dialysis centers. Aquapheresis has been almost exclusively studied as a modality for fluid removal in patients with acute decompensated heart failure who are found to be resistant to incremental doses of intravenous diuretics [67,68]. Nowadays, small size portable devices have been developed for aquapheresis. They are user-friendly machines and are dedicated exclusively for the management of fluid overload (e.g., Aquadex SmartFlow System, Nuwellis Inc., Eden Prairie, MN, USA). They can provide UF rates within a large spectrum (10–500 mL/h), with blood flow rates 40 mL/min. This allows the use of peripheral veins as vascular access and does not mandate admission to an intensive care unit [69,70].

As the theoretical beneficial effects of UF became apparent, with the increasing availability of UF devices, this treatment was widely considered as an alternative to diuretic-based therapy. Several clinical trials have been conducted to examine the efficacy and safety of UF (aquapheresis) in the treatment of ADHF. In Table 3, the most important trials evaluating UF for the treatment of ADHF in comparison to pharmacological therapy are presented. Additional data and comments are given as the Supplementary Material Table S1.

The available trials have enrolled patients with HFrEF and HFpEF and patients with mild to moderate kidney dysfunction and stable blood pressure. Patients with severe kidney impairment and hypotension were typically excluded due to potential complications. These trials include CARESS-HF [75], UNLOAD [73], RAPID-CHF [71], and AVOID-HF [77]. The most recent, prospective, randomized, controlled clinical trial published in 2020 by Hu et al. [79] enrolled 100 patients with ADHF within 24 h of admission randomly assigned into early ultrafiltration (n = 40) or torasemide plus tolvaptan (n = 60) groups. Weight loss and an increase in urine output on days 4 and 8 of treatment were the primary outcomes. Early ultrafiltration resulted in greater weight loss and urine output. On the other hand, readmission and mortality rates were similar between the two groups at the 1-month and 3-month follow-ups.

A new trial, namely REVERSE-HF, started in May 2022 with an expected primary completion date of 30 September 2024, and is still recruiting patients. Nuwellis Inc. is conducting this multicenter, open-labeled, randomized controlled trial to compare ultrafiltration with intravenous diuretics to treat fluid overload in patients with acute heart failure exacerbation [80].

One prospective trial is underway in China to determine the efficacy and safety of ultrafiltration within 24 h of a hospital stay, which also aims to establish a scoring system to guide UF treatment [81]. However, no data have been published so far. Moreover, there are no studies comparing aquapheresis or CRRT with other kidney replacement therapies.

To consolidate the data across several studies, several meta-analyses have been performed [82,83,84,85]. It should be mentioned, however, that these analyses have some limitations that may affect the results and conclusions presented. First, there are considerable differences in intervention protocols, e.g., UF rate, diuretic dosage or timeline of measurement of the studied parameters, as well as heterogeneity of outcomes across the studies. Second, there are possible risks of bias within the studies. Finally, different search terms were used to identify studies during the selection process. This is evident when looking at recently published meta-analyses. A meta-analysis published by Wobbe et al. in 2021 evaluated 8 randomized controlled trials, with 801 participants enrolled [84]. The authors revealed greater fluid removal, significant weight loss, and lower incidence of worsening heart failure and rehospitalizations for heart failure in UF compared to diuretic therapy. There were no differences in renal impairment and all-cause mortality. They concluded that this analysis supports the efficacy of UF therapy without increasing the risk of adverse events. In the meantime, a meta-analysis including 10 clinical trials showed no significant difference in composite endpoint, namely all-cause mortality and all-cause rehospitalizations, between patients undergoing UF compared with those receiving diuretics [86]. All-cause admissions, the need for emergency department visits. and HF-related rehospitalization were similar, just as the risk for hypotension and creatinine rise was not significantly different between the study arms [86]. The authors concluded that UF appeared to be safe but that it did not provide significant benefits compared with diuretic therapy. Finally, a more recently published meta-analysis carried out to investigate efficacy and safety of early UF found that, although early UF is more effective in reducing body weight, it was associated with an increase in serum creatinine and did not reduce the rate of hospital readmissions or mortality [87]. In summary, based on clinical trials and the meta-analyses’ results, there is no evidence to support UF over the pharmacological approach as a first-line therapy in ADHF patients. It should be stressed, however, that the heterogeneity of heart failure patients resulting from different etiologies, distinct phenotypes (HFpEF or HFrEF), and often multi-morbidity burden makes it unlikely that each of these patient subgroups would benefit from a population-based trial. Everyday clinical practice and results from observational cohort studies may suggest that better outcomes could be achieved when therapy is individualized and tailored to a patient’s biological profile and clinical characteristics [88]. Recently, a ten-year study with adjustable UF for the management of ADHF in a real-world cohort has been published [89]. Compared to previously published trials, these patients were older (73 years of age), 52% had HFpEF, worse renal function, and more antecedent HF-related hospitalizations in the year preceding UF therapy. Mean fluid removal with UF was almost 15 L without any impact on kidney function, while at the same time a spectacular decrease in the number of HF rehospitalizations (12.4% at 30 days, 14.9% at 90 days, and 27.3% 1-year post UF treatment) was observed. It is possible that careful patient selection (based on a HF specialty team’s clinical assessment), the adjustment of UF rate during the therapy, and, in general, the individualization of guideline-directed HF therapy, may significantly contribute to these favorable outcomes.

Identification of the subgroup of patients who will benefit most from ultrafiltration, the timing of the initiation and termination of therapy, choosing the most effective protocol of UF and concomitant pharmacologic therapy for HF remain to be determined. Finally, extracorporeal ultrafiltration, despite remarkable progress in the manufacturing of newer, user-friendly UF devices, remains an invasive procedure with potentially serious complications. It requires long-term vascular access and systemic anticoagulation during therapy, with an increased risk of catheter-related complications (clotting or infection) or bleeding requiring transfusion. Moreover, it may be performed exclusively in a hospital environment or even in intensive care units in the case of hemodynamically unstable patients. In addition, the ideal rate of fluid removal is unknown, and it should be individualized and adjusted based on the patient’s renal function, volume status, and hemodynamic status. The initial rate should be based on the degree of fluid overload and the anticipated plasma refill rate from the interstitial fluid [90].

Finally, the cost-effectiveness of UF treatment remains to be established. Whether and to what extent the price of UF supplies and procedures can bring potential savings from a reduced length of stay and readmissions in HF-related hospitalizations must also be assessed [91,92].

## 4. Peritoneal Dialysis

**Figure 6:**
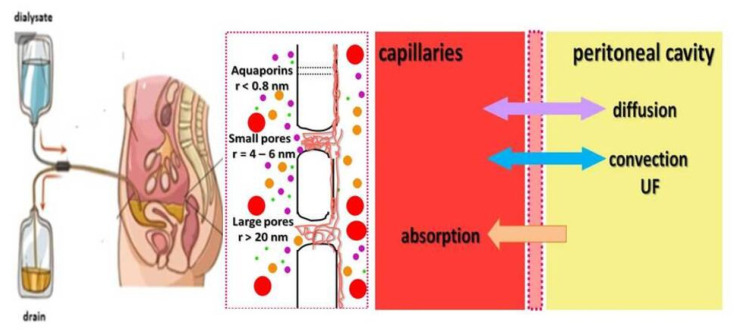
Principles of peritoneal dialysis; modified based on Yu et al. [93].

Peritoneal dialysis (PD) has been used as a home-based RRT for many years. As a continuous modality, it offers the smooth and gentle removal of solutes and water with minimal hemodynamic impact. The principles of PD are depicted in Figure 6.

During peritoneal dialysis, the dialysate is delivered into the peritoneal cavity, left for a dwell period, and then drained. After filling the peritoneal cavity with dialysis fluid, several phenomena are triggered, causing the transport of solutes and water from the blood to the dialysis fluid by diffusion and convection; at the same time, however, there is an absorption process mainly via the lymphatic vessels, which unfortunately reduces, to some extent, the effect of removing water and solutes. According to the three-pore model, transport depends on the relative abundance of large and small pores (endothelial clefts) and ultrapores (aquaporins). Aquaporins are responsible for the transport of free water, through small pores small solutes, e.g., urea and electrolytes can be removed, and large pores enable the removal of solutes and molecules larger than 1000 Da, e.g., inflammatory cytokines.

**Figure 7:**
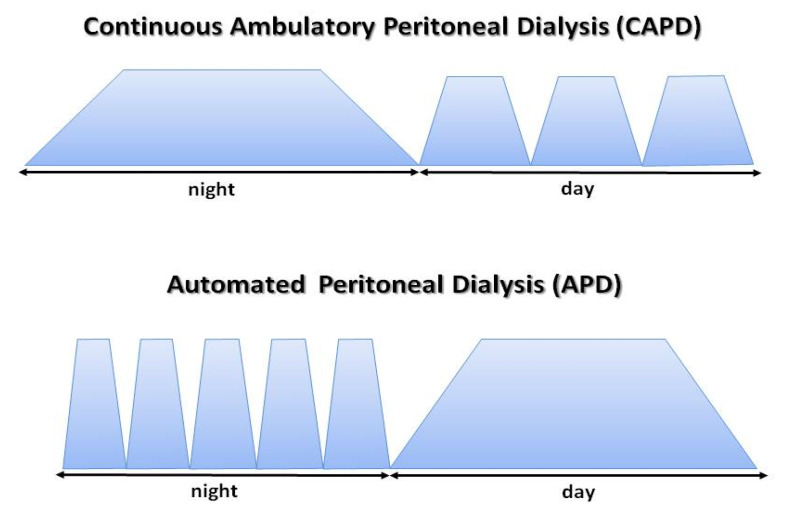
Peritoneal dialysis methods. In continuous ambulatory peritoneal dialysis (CAPD), dialysis fluid exchanges are performed manually by the patient. A typical CAPD schedule includes 2–3 exchanges during the day and 1 at night. Each time, 1.5–2.5 L of dialysis fluid is delivered into the peritoneal cavity. If the exchanges are performed using an automatic device, such as a cycler, this method is called automatic peritoneal dialysis (APD). The patient is connected to the cycler for 8–10 h overnight. Most adult patients treated with APD also require the peritoneal cavity to be filled during the day to achieve good dialysis adequacy. This type of APD is called continuous cyclic peritoneal dialysis (CCPD).

The effectiveness of the dialysis process depends on patient-specific properties of peritoneal membrane; however, it may be widely modified through various methods of dialysis fluid application in terms its volume, frequency of fluid exchanges, and the type and concentration of osmotically active substances [94,95]. Depending on patients’ needs, two PD modalities can be applied, namely continuous ambulatory peritoneal dialysis (CAPD) which means manual exchanges of dialysis fluid, or automated peritoneal dialysis (APD) using devices called cyclers [94], as shown on Figure 7**.**

PD shows significant effectiveness in correcting electrolyte derangements. PD fluids do not contain potassium, and, depending on the dialysis dose, potassium removal can reach up to 50 mmol per day [94,96,97]. It minimizes the risk of hyperkalemia and allows therapy with angiotensin converting enzyme inhibitors, angiotensin receptor blockers, or an aldosterone antagonists without the risk of hyperkalemia. Sodium removal via peritoneal dialysis using glucose-based solutions is better provided by CAPD than APD (150 vs. 90 mmol/day) due to sodium sieving and free water transport (via aquaporins) during the first 60 min of dwell (in CAPD, dwells are longer) while short dwell times in APD are less effective for sodium removal [98,99]. This effect may be abrogated using icodextrin, which induces UF through colloidal osmosis (mainly via small pores not aquaporins), and subsequent removal of 20–50 mmol of sodium. Effective sodium and water removal is necessary to maintain euvolemia, and in HF patients is of paramount importance to achieve decongestion [100,101]. Additionally, a potential benefit of PD may be transperitoneal removal (via convective transport) of inflammatory molecules (MCP-1, tumor necrosis factor α-TNFα, IL-1, IL-6) and so-called cardio-depressants [102,103].

Peritoneal ultrafiltration does not activate the sympathetic nervous system, renin–angiotensin–aldosterone system, and does not stimulate vasopressin and endothelin release which contributes to maintaining diuretics responsiveness [104]. Finally, peritoneal access allows for the controlled drainage of ascites and the maintenance of intra-abdominal pressure at a relatively low level, allowing patients to sustain the response to diuretics and preserve kidney function. Most patients have no contraindications to PD treatment. Absolute contraindications include a history of abdominal surgeries with extensive adhesions, severe inflammatory bowel disease or extensive abdominal wall infection, and patient or caregiver incapability to perform the procedure if assisted PD is not available. A peritoneal catheter may be inserted under general or local anesthesia, or if necessary even at bedside via a minimally invasive percutaneous procedure, and can be used immediately after implementation.

**Figure 8:**
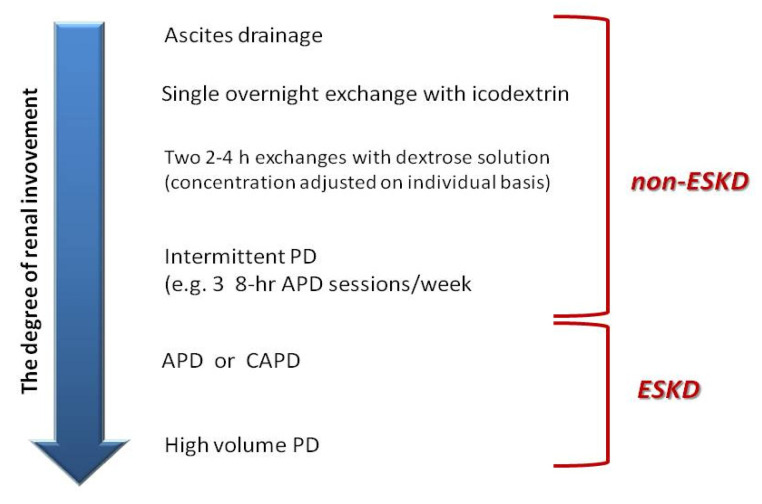
Peritoneal ultrafiltration and peritoneal dialysis methods depending on the degree of kidney damage. Courtesy of J Matuszkiewicz-Rowińska.

There is growing evidence that peritoneal dialysis, with its flexibility in techniques, regimens, and solutions, may be a viable therapy option for patients with cardiorenal syndrome and refractory volume overload. There are two patient groups with cardiorenal syndrome who could benefit from PD—patients with non-end-stage kidney disease (non-ESKD) and those with ESKD. In the first group, PD is primarily used for the relief of refractory congestion and is modified depending on the patients’ needs for fluid removal and/or solute clearance (Figure 8).

PD or peritoneal ultrafiltration (PUF) have been used successfully in HF patients for years; however, the published data come from observational studies with rather small and mixed populations, as well as different PD schedules in terms of the method (CAPD or APD), kind of solutions, and number of dwells. Attempts to conduct randomized controlled trials have failed due to inadequate patient recruitment. Barriers to recruitment included frailty, unwillingness to engage in invasive therapy, and probably suboptimal coordination between cardiology and nephrology services. In Table 4, selected studies of PD or PUF in HF and CRS are presented. Additional data and comments are given as the Supplementary Materials Table S2.

To obtain consistent evidence on the effectiveness and safety of PD/PUF in HF and CRS, several meta-analyses have been performed [123,124,125,126,127]. Most studies revealed spectacular (about 80%) decreases in hospitalizations during the PD/PUF treatment in terms of the number, length, and the total days of hospitalization/patient/year (34.8 day/pt/year) [123,124,125,127]. It is considered the most significant effect of the implementation of the PD/PUF treatment due to its effective decongestion and improved reactivity to diuretics.

The mechanisms involved in these beneficial effects are associated with excess fluid and sodium removal, decreased stimulation of the renin–angiotensin–aldosterone axis and sympathetic nervous system, and a reduction in intra-abdominal pressure. In some subgroups of patients, especially those with right heart failure, effective drainage of ascites via a Tenckhoff catheter may be associated with clinical (NYHA class and hospitalization reduction) and kidney function improvement [121,128,129]. This may be a beneficial solution, especially in patients who require frequent large-volume paracenteses. After some time, as kidney disease progresses, peritoneal drainage alone becomes insufficient, and it is necessary to start regular dialysis exchanges [121,129]. The implementation of the PD/PUF causes not only excessive fluid removal, but also sufficient and effective solute (e.g., sodium and potassium) clearance. This allows for the optimization of the pharmacological treatment for heart failure. Unfortunately, in published studies, the data on pharmacological treatment are very scarce, and it is not possible to confirm to what extent the change in the clinical outcome is attributable only to PD/PUF and to what extent this change is due to the combined effect of optimized guideline-directed pharmacotherapy and PD/PUF. In most studies, implementation of PD/PUF was associated with a rapid, significant, and long-term improvement in clinical status, as demonstrated by a reduction in NYHA functional class. NYHA class decreased by at least one (−1.37; 95% CI: −0.78 to −1.96, *p* < 0.0001) [123,124,125,126,127].

The clinical improvement may be associated with the improvement in cardiac function. Most studies reported LVEF before and after intervention, and a statistically significant improvement was reported (4.33%, 95% CI: 1.88–6.78%, *p* < 0.0001) [124,125,127]. Indeed, excess fluid removal via ultrafiltration may improve cardiac output due to changes in the Frank–Starling curve. Moreover, the removal of some cytokines or humoral factors with specific myocardial depressant activity via peritoneal membrane has been proposed as a potential explanation for this favorable effect. Nevertheless, the evidence is very limited, and large-scale studies are needed [118].

Right heart function has been assessed rarely. Pulmonary artery pressure (PAP) has been assessed by echocardiography in only a few reports. In most of them, a statistically significant decrease in PAP was reported [110,113,115,119].

An important outcome assessed in most studies was the impact of PD/PUF on kidney function. In ESKD patients starting KRT, using PD favors longer maintenance of residual kidney function. Pooled results from HF studies showed a very small and not significant decrease in glomerular filtration rate (GFR) after PD/PUF implementation (−3.0 mL/min, 95% CI: −6.0 to 0, *p* = 0.05) [124,125,127]. Only a few studies reported urinary volume and diuretics doses. Increased or at least stable urine output was observed, while diuretic doses were reduced [109,111,120,121,122]. It is worth noting that the heterogeneity of the studies may be an important factor in the assessment of the impact of PD/PUF on kidney function. In some studies, obligatory extracorporeal UF (or HD) to relieve congestion before starting PD/PUF was applied and the duration of follow up was quite variable, which may affect the results [105,108,109,110,113,114,119]. When PD/PUF is started as a first-line therapy, increased diuresis may be associated with the improvement of kidney function (6–12 months). Later on, kidney function gradually worsens and PD/PUF prescription must be tailored to maintain effective solute clearance and fluid removal [109,111,115,121]. It appears to be unrelated to the PD/PUF procedure, but rather to the specific characteristics of this group, namely pre-existing nephropathy, age, and multi-morbidity. Symptomatic improvement has a favorable effect on the quality of life; however, it was formally assessed using appropriate scoring tools in only a few studies [110,112].

Because most studies included in the meta-analyses had cohort or retrospective designs, it is impossible to determine the impact of the procedure on survival. In addition, historical controls were used as comparators, and the life expectancy of patients with severe HF from other studies or life expectancy arising from comorbidity burden were assessed for comparisons. All-cause mortality at 1 year (from 17 studies) has been estimated at 37%; however, some studies show a remarkable survival benefit for PD/PUF-treated patients, with rates as high as 90% at 1 year [127].

The rate of peritoneal dialysis complications (mechanical and infectious) is low, and comparable to what is observed in standard PD programs in patients with ESKD. Most common infectious complications (peritonitis or catheter infection) are generally easily treatable without the need to interrupt PD/PUF [124,125,127].

The cost-effectiveness of the procedure should be also mentioned. It seems quite obvious that the implementation of home-based therapy and a significant reduction in HF-related hospitalizations, in particular in patients group requiring frequent hospitalizations, favors a reduction in total costs. Nevertheless, formally, the data demonstrating the cost-effectiveness of the procedure are limited. In one Spanish study, it was shown that costs from the PD/PUF procedure were lower than continuation of conventional therapies, with a saving of about EUR 11,000 per patient [110].

## 5. When Should Ultrafiltration Be Considered in HF Patients?

**Figure 9:**
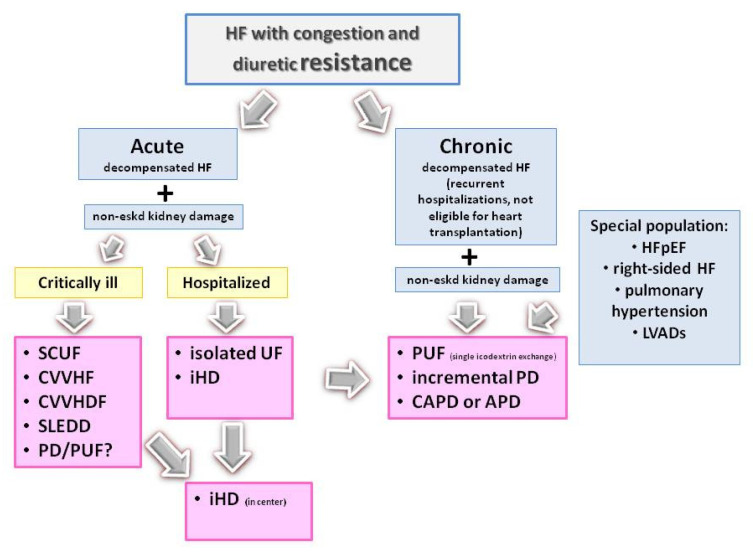
Algorithm for the selection of kidney replacement therapy modality in HF patients with refractory congestion and diuretic resistance.

Precise identification of patients who will benefit from extracorporeal or peritoneal ultrafiltration remains a challenge. There is also inadequate evidence comparing PD versus extracorporeal techniques. The current European and American guidelines for the management of HF suggest that KRT may be considered in refractory volume overload, which is unresponsive to diuretic therapy, without distinguishing between types of KRT [30,130]. In a recently published scientific statement from the American Heart Association, it was indicated that PD may be an attractive modality of dialysis therapy for HF patients [50]. There are, however, no robust data to establish indications for the choice of extracorporeal or peritoneal techniques. It seems reasonable to suggest that the KRT type should be adapted to the clinical characteristics of the patients. A proposed algorithm for selecting a KRT technique is presented on Figure 9.

According to this algorithm, extracorporeal kidney replacement therapy techniques may be feasible in hospitalized patient with acute decompensated HF, such as continuous therapies in hemodynamically unstable patients and intermittent in other patients. Nevertheless, the positive experience with acute start of PD with an emergent indication in acute kidney injury, and more recently in severe septic condition during SARSCoV-2 virus infection, suggests that HF patients could also benefit from this kind of therapy [131,132]. This should, however, be confirmed in large-scales studies focused on this patient populations.

Peritoneal dialysis or peritoneal ultrafiltration as home-based continuous therapy seems to be an attractive and viable option for long-term treatment. It could be offered to patients with refractory congestion and diuretic resistance despite optimal pharmacological therapy, those with frequent HF-related hospitalizations, and those not eligible for heart transplantation as a part of end-of-life care. PD-based therapy should also be considered in patients with right-sided HF, pulmonary hypertension, or in individuals with left ventricular-assisted devices (LVADs) or other cardiac devices to reduce the risk of vascular access-related infections [50].

## 6. Conclusions

Cardiorenal patients are fragile and are at increased risk for both decompensation of heart failure and worsening kidney function. Careful follow-up and patient-centered treatment may be crucial to improving heart and kidney outcomes. Patients would likely benefit from a close collaboration between cardiologists and nephrologists when deciding on treatment modalities and care [133].

There is an urgent need for clinical trials to obtain high-quality data for goal-directed medical therapy in chronic CRS with moderate to severe decline in kidney function and to develop best clinical practice guidelines. This concerns the clarification of diagnostic strategies (imaging modalities, cardiac and renal biomarkers), the establishment of effective and safe pharmacological treatment protocols, device therapies, and indications, the optimal moment of initiation, and the most appropriate kidney replacement therapy methods.

## Figures and Tables

**Table 1:** Comparison of different kidney replacement therapy methods.

Modality	Solute Transport	Duration (h)	Blood Flow (mL/min)	Dialysate Flow (mL/min)	Fluid Removal Rate (mL/h)	Advantages	Disadvantages
SCUF	Convection	≥24	50–100	-	0–300	Slow, sustained fluid removal; hemodynamic stability	Immobilization
IUF	Convection	3–5	200–350	-	0–2000	Shorter procedure	Higher risk of hemodynamic instability
IHD	Diffusion	3–5	200–350	300–800	0–1000	Fast small solute and fluid removal, effective control of toxemia and volemia	Higher risk of hemodynamic instability, fluctuating fluid balance
SLEDD	Diffusion	6–16	100–300	200–300	0–500	Slower solute and fluid removal, effective control of toxemia and volemia; hemodynamic stability	Worse than in convective techniques for large and mid-size molecule removal
CVVHF	Convection	≥24	50–100	-	0–300	Slow, sustained solute and fluid removal; large and mid-size molecule removal; hemodynamic stability	Immobilization, anticoagulation mandatory, high staff involvement, high costs
CVVHDF	Convection + diffusion	≥24	50–100	20–40	0–300	Slow, sustained solute and fluid removal; large and mid-size molecule removal + small solute removal; hemodynamic stability	Immobilization, anticoagulation mandatory, high staff involvement, high costs

SCUF—slow continuous ultrafiltration, IUF—isolated ultrafiltration, IHD—intermittent hemodialysis, SLEDD—slow low-efficiency extended (daily) dialysis, CVVHF—continuous veno-venous hemofiltration, CVVHDF—continuous veno-venous hemodiafiltration.

**Table 2:** Potential advantages of extracorporeal ultrafiltration in HF.

Rapid and adjustable fluid removal and improvement in symptoms of congestion
Higher mass clearance of sodium
Lack of neurohormonal activation (SNS, RAAS, and AVP) with long-lasting beneficial effect on the neurohormonal axis
Decreased risk of electrolyte abnormalities, e.g., hypokalemia
Reduction in renal venous congestion with an improvement in renal hemodynamics
Improvement in diuretic resistance, urine output, and natriuresis
Decreased hospital length of stay and rate of HF-related hospitalizations

RAAS—renin–angiotensin–aldosterone system, SNS—sympathetic nervous system, AVP—arginine vasopressin.

**Table 3:** Selected trials evaluating extracorporeal UF for the treatment of acute decompensated HF.

Study/ Reference	Pts	Intervention	Control	Follow-Up	Key Findings
RAPID-CHF (RCT) 2005 [71]	40	Single, 8 h course of UF max rate 500 mL/h; diuretics held during the 8 h of UF	Standard CHF therapies	30 days	↔ weight loss at 24 h, ↓ dyspnea and CHF symptoms significantly improved, ↔ length of hospital stay
EUPHORIA (single arm) 2005 [72]	20 ADHF, Scr ≥ 1.5 mg/dL, or diuretic resistance	UF max rate 500 mL/h; if SBP fell to ≤80 mm Hg, UF rate reduced to 200 mL/h	N/A	90 days	Fluid removed 8654 ± 4205 mL; significant improvement in clinical signs and symptoms of volume overload ↓ NYHA class
UNLOAD (RCT) 2007 [73]	200 ADFH, signs and symptoms of congestion	During the first 48 h UF ≤ 500 mL/h; no diuretics; sodium 2 g/d, fluid intake 2 L/d	Sodium 2 g/d, fluid intake 2 L/d; intravenous diuretics ending at 48 h after randomization	90 days	greater weight loss; ↔ dyspnea score improvement ↓ patients’ rehospitalizations, rehospitalization days ↓ unscheduled visits
ULTRADISCO (RCT) 2011 [74]	30 ADFH, signs and symptoms of congestion	UF 100–300 mL/h adjusted to SBP and HR; no diuretics and vasoactive drugs	Continuous infusion of furosemide 250 mg/24 h adjusted to SBP and HR; no vasoactive drugs	36 h	↑ stroke volume index cardiac index, cardiac power; ↓ systemic vascular resistance, NTproBNP, aldosterone ↓ sign and symptom score
CARRESS-HF (RCT) 2012 [75]	188 ADHF, worsening kidney function, persistent congestion	UF rate 200 mL/h; no diuretics; vasoactive drugs only as rescue therapy	Stepped pharmacologic-therapy: diuretics to maintain urine output of 3–5 L/d, vasoactive therapy on the individual patient’s needs	60 days	↑ serum creatinine level in UF group; ↔ weight loss
CUORE (RCT) 2014 [76]	56 HF—NYHA class III or IV, LVEF < 40%’ >4 kg weight gain/2 months	1 or 2 sessions of UF 100–500 mL/h; cumulative fluid removal of >2 L (no more than 75% of the estimated weight increase	IV diuretics, standard of care	1 year	↓ rehospitalizations in UF group; ↑ freedom from rehospitalization for HF in UF group
AVOID-HF (RCT) 2016 [77]	224 ADHF; fluid overload on oral loop diuretics	Adjustable UF (138 ± 47 mL/h); restriction in sodium and fluid intake; vasoactive drugs only as rescue therapy	Diuretics to maintain urine output of 3–5 L/d, restriction in sodium and fluid intake; vasoactive drugs only as rescue therapy	90 days	↔ days to first HF event
Hanna et al. (RCT) 2012 [78]	36 ADHF, NYHA class III and IV, LVEF <40%, mean PCWP > 20 mm Hg	UF rate 400 mL/h for 6 h decreased to 200 mL/h; no diuretics, vasoactive medications at the physician’s discretion	IV diuretics designated by the treating clinician; vasoactive medications at the physician’s discretion	90 days	↓ time to primary endpoint in UF group
Hu et al. (RCT) 2020 [79]	100 ADHF, volume overload	UF (200–300 mL/h) day 1–3; torasemide + tolvaptan days 4–7.	Torasemide + tolvaptan	90 days	↑ weight loss and ↑ urine in UF group

↓ decrease; ↔ no effect; ↑ increase.

**Table 4:** Selected studies evaluating peritoneal dialysis or peritoneal ultrafiltration for the treatment of HF.

Reference	Pts Age (y)	eGFR/S_Cr_ (mL/min/mg/dL)	NYHA Class	LVEF (%)	Observation (Months)	1-Year Survival	Key Findings
Gotloib 2005 [105]	20 65.7 ± 7.6	14.8 ± 3.8	IV	31.2 ± 4.7	19.8 ± 7.37	90	↓ NYHA class, ↑ LFEF, ↓ hospitalization
Diez Ojea 2007 [106]	5 60 ± 6.3	43.6 ± 27.07	IV	35	13.8 ± 5.6	N/A	↓ NYHA class, ↑ LFEF, ↓ hospitalization
Basile 2009 [107]	4 71.5 ± 5.6	Scr 3.55 ± 1.12	IV	45 ± 27.7	24.3 ± 15.6	N/A	↓ NYHA class, ↑ LFEF, ↓ hospitalization
Cnossen 2010 [108]	24 67 ± 10	14.8 ± 12.1	N/A	33 ± 16	1.03 ± 0.84 years	N/A	↔ LFEF, ↓ hospitalization
Nakayama 2010 [109]	12 81 ± 6	10.5 ± 8.2	III and IV	56 ± 10	Median 26.5	N/A	↓ NYHA class, ↔ LFEF, ↓ hospitalization
Sanchez 2010 [110]	17 64 ± 9	35 ± 6	III and IV	33.3	15 ± 9	82	↓ NYHA class, ↑ LFEF, ↓ hospitalization
Sotirakopoulos 2011 [111]	19 71.3 ± 8.1	23.8 ± 10.6	III and IV	28.6 ± 8.6	median 16	68	↓ NYHA class, ↑ LFEF, ↓ hospitalization
Nunez 2012 [112]	25 75.1 ± 8.2	Median 33	III and IV	40 ± 14	Median 14	N/A	↓ NYHA class, ↔ LFEF, ↓ hospitalization
Ruhi 2012 [113]	6 72.8 ± 4.9	49 ± 14.6	III and IV	28.2 ± 4.5	6–36	N/A	↓ NYHA class, ↔LFEF, ↓ hospitalization
Koch 2012 [114]	118 73.2 ± 11.4	19.2 ± 13.3	III and IV	Median 43.5	1.11 ± 1.17 years	55	↓ NYHA class, ↑ LFEF, ↓ hospitalization
Bertoli 2014 [115]	48 74 ± 9	21 ± 10	II, III, and IV	30 ± 11	24	85	↓ NYHA class, ↑ LFEF, ↓ hospitalization
Courivaud 2014 [116]	126 72 ± 11	33.5 ± 15	N/A	38 ± 19	16 ± 16	58	↑ LFEF, ↓ hospitalization
Frolich 2015 [117]	39 67 ± 11	Median 22	III and IV	24 ± 7	9.5	67	↓ NYHA class, ↔ LFEF, ↓ hospitalization
Hedau 2018 [118]	30 62.3 ± 7.5	Scr 3.18 ± 0.98	III and IV	29.3 ± 7.4	6	N/A	↓ NYHA class, ↑ LFEF, ↓ hospitalization
Pavo 2018 [119]	40 65	Median 19.4	N/A	Median 29	12.3	55	↓ hospitalization
Shao 2018 [120]	14 53.6 ± 15.4	27.8 ± 9.87	III and IV	24.6 ± 3.78	6	N/A	↓ NYHA class, ↔ LFEF, ↓ hospitalization
Wojtaszek 2019 [121]	15 72 ± 9	32 ± 11	III and IV	34.3 ± 12.4	24	93	↓ NYHA class, ↑ LFEF, ↓ hospitalization
Grossekettler 2019 [122]	159 72.8 ± 12.1	24 ± 11.3	II, III, and IV	31 ± 13	n.a.	61	↓ NYHA class, ↑ LFEF, ↓ hospitalization

↓ decrease; ↔ no effect; ↑ increase.

## Data Availability

No new data were generated.

## Supplementary Materials

The following supporting information can be downloaded at: https://www.mdpi.com/article/10.3390/ijms26062456/s1.
