# Supplementary material for: Heart Failure—Focus on Kidney Replacement Therapy: Why, When, and How?
Source: Int J Mol Sci. 2025 Mar 10;26(6):2456. doi: 10.3390/ijms26062456 (PMC11941842; doi:10.3390/ijms26062456)
Supplement: Supplementary file 1 [file ijms-26-02456-s001.zip › ijms-3473757-supplementary.pdf]

Table S1. Selected trials evaluating extracorporeal UF for the treatment of acute decompensated HF.

Study/ Reference	Additional informations and comments
**RAPID-CHF (RCT) 2005 (30)**	UF well tolerated,<br>one death in the UF group (unrelated to UF)
**EUPHORIA (single arm) 2005 (31,32)**	↔ kidney function and blood pressure<br>improved quality of life.<br>↓ length and number of hospitalizations; positive effect for 3 months
**UNLOAD (RCT) 2007 (33)**	↔ rises in serum creatinine levels<br>lengths of hospitalization comparable;<br>NYHA class, MLWHFQ , 6-min walk distance, similarly improved
**ULTRADISCO (RCT) 2011 (34)**	↓serum creatinine<br>↔ BP and HR;<br>significant NYHA class improvement
**CARRESS-HF (RCT) 2012 (35)**	clinical decongestion at 96 hours low in two study arms;<br>↔ scores on the dyspnea and well being scale<br>serious adverse events higher in UF arm
**CUORE (RCT) 2014 (36)**	↔ hospitalization length , ↔ weight reduction at discharge<br>stable clinical condition during follow up in UF group<br>↔ 1-year mortality
**AVOID-HF (RCT) 2016 (37)**	↓ HF and cardiovascular events in UF group, ↔ kidney function changes<br>↔ the 90-day mortality<br>↑adverse events in UF group
**Hanna et al (RCT) 2012 (38)**	↑ weight reduction, ↑ total volume removed, ↓ hospital length in UF group<br>↔ kidney function, biomarkers,
**Hu et al (RCT) 2020 (39)**	↓ NYHA class, JVP score, IVC diameter and BNP level in UF group,<br>↔ readmission and mortality rates;<br>↔ kidney function

Table S2. Table 4. Selected studies evaluating peritoneal dialysis or peritoneal ultrafiltration for the treatment of HF.

Reference	Additional informations and comments
**Gotloib 2005 (62)**	CCI 7.8±1.8, CVVHF before PD, ↓ thoracic fluid conductivity in bioimpedance Peritonitis 0.27 episodes/patient/year
**Diez Ojea 2007 (63)**	CCI 6±1 ↓pulmonary hypertension icodextrin single exchange or in CAPD prescription
**Basile 2009 (64)**	single overnight exchange with icodextrin ↑ diuresis increase ↑ kidney function no peritonitis episode
**Cnossen 2010 (65)**	HD before PD incremental PD with icodextrin
**Nakayama 2010 (66)**	CVVHF before PD, incremental PD with icodextrin , ↔ kidney function ↓ diuretics doses, ↓ BNP, aldosterone and plasma renin activity
**Sanchez 2010 (67)**	CCI 6.9±1.7 extracorporeal UF before PD , single overnight exchange with icodextrin ↓ PASP, ↑ quality of life, peritonitis 0.02/patient/year, reduced total health care costs
**Sotirakopoulos 2011 (68)**	PD with icodextrin ↔ kidney function, ↑ diuresis ↓ furosemide dose peritonitis 2 pts
**Nunez 2012 (69)**	CCI 5±2, significant improvement in quality of life ↑ 6 min walk test, peritonitis 0.75/patient/year, leaks 3pts
**Ruhi 2012 (70)**	CVVHF before PD single overnight exchange with icodextrin ↔ kidney function ↓ PASP no PD complications
**Koch 2012 (71)**	HD before PD peritonitis 0.053/patient/year, catheter dysfunction 0.084/patient/year
**Bertoli 2014 (72)**	single overnight exchange with icodextrin or PD with icodextrin ↔ kidney function ↓ PASP
**Courivaud 2014 (73)**	peritonitis 1 episode in 26.2 patient-moths
**Frolich 2015 (74)**	↓ residual renal function, ↓ urinary volume ↑ quality of life
**Hedau 2018 (75)**	↑ kidney function ↓ IL-1, IL-6 and TNFα levels
**Pavo 2018 (76)**	CVVHF or HD before PD, 35% started treatment only with ascites drainage

	↓ sPAP
**Shao 2018 (77)**	↑ urine volume<br>↔ kidney function<br>↓ BNP level
**Wojtaszek 2019 (78)**	CCI 9±1.2<br>single overnight exchange with icodextrin
**Grossekettler 2019 (79)**	↔ kidney function, ↔ urine volume
